# Approaches Involved in the Vegetable Crops Salt Stress Tolerance Improvement: Present Status and Way Ahead

**DOI:** 10.3389/fpls.2021.787292

**Published:** 2022-02-21

**Authors:** Tusar Kanti Behera, Ram Krishna, Waquar Akhter Ansari, Mohd Aamir, Pradeep Kumar, Sarvesh Pratap Kashyap, Sudhakar Pandey, Chittaranjan Kole

**Affiliations:** ^1^ICAR-Indian Institute of Vegetable Research, Varanasi, Varanasi, India; ^2^ICAR-Directorate of Onion and Garlic Research, Pune, India; ^3^ICAR-Central Arid Zone Research Institute, Jodhpur, India; ^4^National Institute for Plant Biotechnology, New Delhi, India

**Keywords:** oxidative stress, physio-biochemical responses, antioxidant, transgenic crops, gene regulation, yield loss

## Abstract

Salt stress is one of the most important abiotic stresses as it persists throughout the plant life cycle. The productivity of crops is prominently affected by soil salinization due to faulty agricultural practices, increasing human activities, and natural processes. Approximately 10% of the total land area (950 Mha) and 50% of the total irrigated area (230 Mha) in the world are under salt stress. As a consequence, an annual loss of 12 billion US$ is estimated because of reduction in agriculture production inflicted by salt stress. The severity of salt stress will increase in the upcoming years with the increasing world population, and hence the forced use of poor-quality soil and irrigation water. Unfortunately, majority of the vegetable crops, such as bean, carrot, celery, eggplant, lettuce, muskmelon, okra, pea, pepper, potato, spinach, and tomato, have very low salinity threshold (EC_t_, which ranged from 1 to 2.5 dS m^–1^ in saturated soil). These crops used almost every part of the world and lakes’ novel salt tolerance gene within their gene pool. Salt stress severely affects the yield and quality of these crops. To resolve this issue, novel genes governing salt tolerance under extreme salt stress were identified and transferred to the vegetable crops. The vegetable improvement for salt tolerance will require not only the yield influencing trait but also target those characters or traits that directly influence the salt stress to the crop developmental stage. Genetic engineering and grafting is the potential tool which can improve salt tolerance in vegetable crop regardless of species barriers. In the present review, an updated detail of the various physio-biochemical and molecular aspects involved in salt stress have been explored.

## Introduction

Nearly, three thousand species of plants are being utilized for the food by human; anyhow, presently, the total global population mainly depends mostly upon 20 species of crops for its major calorie needs from which 50% is contributed by eight cereal crop species (Krishna et al., 2019). The insufficient availability of vegetables is mainly due to increasing population, abiotic (drought, salt, heat, water logging, etc.) and biotic (virus, viroids, bacteria, fungi, nematodes, and insects) stresses, which potentially reduce the production and quality of the vegetable crops (Ingram, 2011; Prasanna et al., 2015; Karkute et al., 2019; Krishna et al., 2021a,b; Singh et al., 2021; Soumia et al., 2021). The key challenge of modern agriculture is to fulfill the nutritional and food security of the global growing population. Among abiotic stresses, salt stress is the second most destructive stress as it persists throughout the crop life cycle. Salinity stress is one of the most important environmental constraints that limits the economic productivity of vegetable crops (Hong et al., 2021). Salinity in soil is influenced by a regular fluctuation in climatic conditions, irrigation of crops with low quality water, excessive use of ground water, and massive introduction of irrigation associated intensive farming (Tiwari et al., 2010). Further, prolonged water stress conditions in soil could result in increased salinity in soil profile due to lack of leaching rain, increased bore water salinity, and evaporation from irrigation dams. Vegetable crops are more prone to climatic changes compared with other horticultural crops (Giordano et al., 2021), and particularly, salinity stress influences the growth and development throughout their ontogeny. Salinity-induced oxidative stressed in vegetables could affect the qualitative and quantitative value of vegetables as this oxidative stress could lead to a plethora of biochemical and physiological changes in plants (Kashyap et al., 2020, 2021). The most common of them include membrane damage, leakage of substances causing water imbalance and plasmolysis, disturbance in ROS detoxification system, changes in nutrient flux and dynamics, and photosynthetic attributes. These changes ultimately affect the physiological activities like respiration, photosynthesis, transpiration, hormonal regulation, water use efficiency, germination, production of antioxidants, and plasma membrane permeability (Chourasia et al., 2021). The most common approach adopted by plants during such extreme conditions is transcriptional reprogramming of stress responsive genes (Aamir et al., 2017; Tolosa and Zhang, 2020), although the conventional breeding approaches have helped a lot in developing stress-tolerant breeds of vegetables. However, we do not still have developed optimum solutions to prevent the economic losses of vegetables from salt stress, particularly, in intensively irrigated areas (Machado and Serralheiro, 2017). Transgenic technology for salt stress tolerance has been reported as one of the most crucial tool in developing the stress-tolerant vegetable crops (Kumar et al., 2017). For example, to avoid salt tolerance in plants, genes encoding for proteins like Na^+^ “exclusion” (PM-ATPases with SOS1 antiporter, and HKT1 transporter), vacuolar compartmentalization of Na^+^ V-H^+^-ATPase and V-H^+^-PPase with NHX antiporter, and also other genes encoding proteins such as aquaporins and dehydrins that are involved in mitigation of water stress during salinity have been transferred and/or overexpressed in tomato or *Arabidopsis* through transgenic technology (Kotula et al., 2020). Since tomato is one of the most important vegetable crop and experimental model for molecular biology studies, most of the research done so far with respect to abiotic and biotic stresses have been done in tomato (Meena et al., 2016, 2018; Zehra et al., 2017a,b). For example, overexpression of *LeNHX2* and *SlSOS2* proteins resulted in salinity tolerance in tomato transgenic lines (Maach et al., 2021). The stress-responsive genes expressed during salinity stress and their fine-tuning could be an eminent tool for developing stress-resistant varieties.

On an average every year approximately 12 billion USD are lost worldwide due to the salinity stress which greatly affects the agriculture production (Zahedi et al., 2019). Almost 10% of the world’s entire land area (950 Mha), 20% of the world’s cultivated land (300 Mha), and approximately 50% of the total irrigated land (230 Mha) are consequently distressed with extreme salinity (Abiala et al., 2018).

## Salt Stress Responses in Plants

Important physiological and biochemical processes in plants are adversely affected by salinity in various ways through an intense concentration of salts and unavoidably leading to a gradual reduction in plant growth. High salt concentration in rhizosphere of plant cell causes osmotic effect, which remains as a chief contributor to growth reduction during the preliminary stages of a plant life cycle. Amendment in K^+^/Na^+^ ratio arises when ions reach the plant cell through saline water, leading to augmented Na^+^ and Cl^–^ ion, inflicting extensive damage of numerous physiological processes like protein metabolism and enzyme activities (Tester and Davenport, 2003). The interactions between salts and essential mineral nutrients may consequently result in significant nutrient deficiencies and disproportion. Ionic imbalances may also result in decreased uptake of various significant minerals like potassium, manganese, and calcium to the plants. However, in response to ionic and nutrient imbalances, salt-tolerant plants have uniquely developed the capability of accumulation and compartmentalization of Na^+^ and Cl^–^ in their matured leaves, but sensitive species at absurdly high salinity stage cannot manage to compartmentalize the ions or Na^+^ transport, leading to the ionic or osmotic effect. Considerable reduction in plant height has been documented under different abiotic stresses. Due to salinity, plants are exposed to serious water deficit conditions that reduces the leaf growth and leaf areas in several species such as wheat (Sacks et al., 1997), poplar (Wullschleger et al., 2005), and cowpea (Manivannan et al., 2007). One example of the physiological changes in response to salt is shedding of the older leaves of plants (Shao et al., 2008). The upsurge in root to shoot ratio due to salinity conditions was found to be associated with the ABA content of plants (Sharp and LeNoble, 2002). Plant productivity under salinity is strongly correlated with biomass distribution.

## Mechanism of Salinity Tolerance

Salinity tolerance is related to a list of morphological, biochemical, molecular, and physiological traits that govern the plant growth and productivity (Alexieva et al., 2001). Morphological and physiological adaptation toward tolerance to the salt-induced osmotic stress is also facilitated by reducing water loss from cuticle and stomata and maximized uptake of water by root to maintain the osmotic adjustment (Rai et al., 2021). Tolerance and adaptation to salt stress are governed by a cascade of molecular networks, which trigger response processes like production of stress proteins, upregulation of antioxidants, and accumulation of compatible solutes (Nahakpam and Shah, 2011) to provide homeostatic reestablishment of cells and to repair and protect the damaged membranes and proteins. On the basis of responses to salinity, plants are categorized as either halophytes or glycophytes (Flowers and Flowers, 2005). Under high saline conditions, glycophytes are unable to survive, whereas halophytes can easily grow and reproduce. Tissue tolerance and salt avoidance are two main approaches implemented by plants to overcome the salt stress. Plants also execute the compartmentalization of ions in the plant tissues. To regulate their osmotic pressure, plants continuously generate water-soluble and low molecular-weight compatible solutes like sugars, glycinebetaine, and proline and the metabolic processes of plants are not disturbed. Plants also produce many enzymatic and non-enzymatic antioxidants to minimize the adverse effect of salinity. In the procedure of plant tissue tolerance, ions compartmentalization occurs in the vacuole, resulting in sustained salt concentration in cytosol, and thus the cytoplasm of the plant cell can be protected from water stress and ion toxicity (Chinnusamy et al., 2005).

To cope with salt stress many strategies have been evolved and developed to secure the vegetable yield under salt stress like transgenic development, regulation of transcription factors (TFs), and grafting. In this review, we have presented updated information on biotechnological interventions in vegetable crops for salt stress.

## Transgenic Vegetables for Salt Stress Tolerance

**FIGURE 1:**
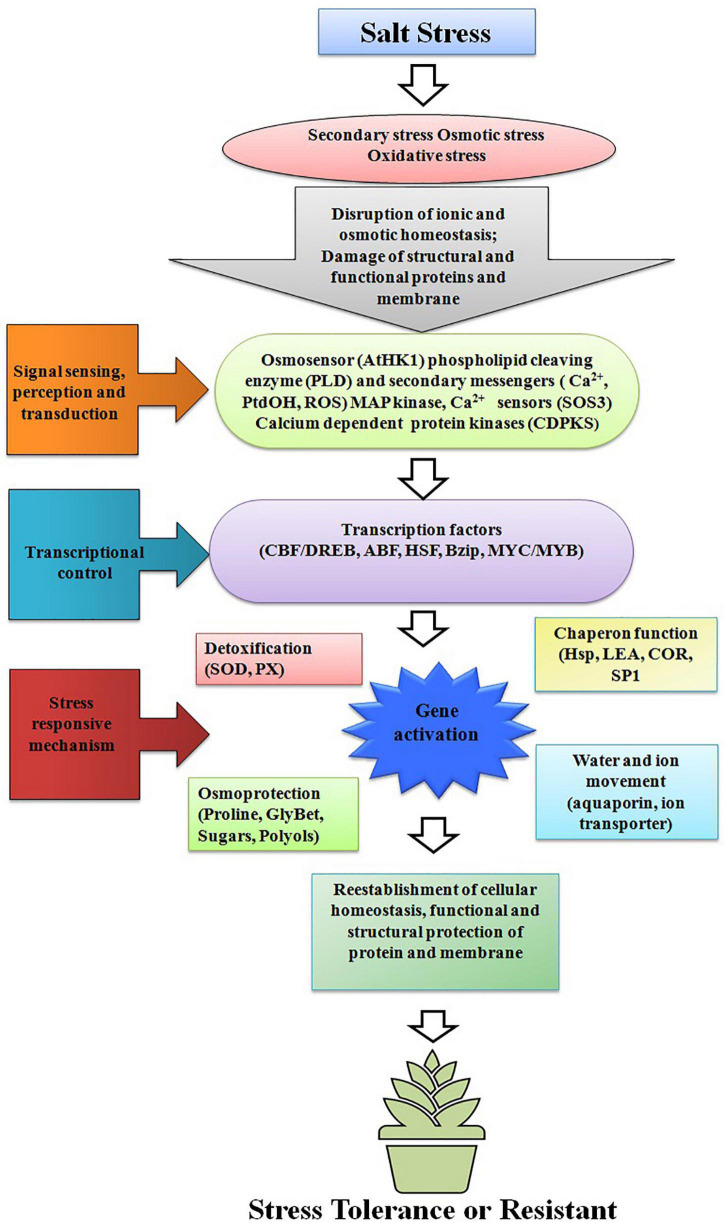
Mechanisms of transgene action in transgenic plants; downstream signaling process and transcription controls that stimulates stress-responsive mechanisms to reestablish cellular homeostasis and damage repair.

Vegetables are the cheapest source of minerals, vitamins, antioxidative phytochemicals, and consumed all over the world in raw, semicooked, cooked, and/or in processed forms. Salt stress does not affect vegetable yield but it also affects the nutritional quality of the vegetables. Due to lack of novel salt tolerance in the gene in many vegetable crops gene pools, the transgene has been transferred from the non-parent sources like bacteria, fungi, plant, and animals. For developing transgenic vegetable crops, firstly gene is being identified, characterized, then transferred in the desired vegetables for salt stress. The transgene is being induced under salt stress and the upregulation or downregulation of transgenes initiates a cascade of stress regulatory phenomenon, which ultimately results in salt tolerance (Figure 1). Among vegetable crops, Solanaceous crops like potato, tomato, capsicum, and chili constitute major group of vegetables consumed all over the world, and out of these, potato is the most important and ranks third in the world in terms of economic importance and a key agriculture crop for food and nutritional security. Potato is cultivated globally and very sensitive to salt stress, and more than 60% crop loss is caused abiotic stresses including salt (Upadhyaya et al., 2011; Xu et al., 2014; Shafi et al., 2017). To improve potato yield and quality under salt stress condition, many transgenic potato plants have been developed using different genes with different modes of action (Shafi et al., 2017; Wang et al., 2019; Ali et al., 2020). Many osmoprotectant genes like *P_5_CS, mtlD*, and *AtBADH* have been transferred to potato, which significantly improves the salt tolerance under salt stress (Karthikeyan et al., 2011; Rahnama et al., 2011; Zhang et al., 2011). Like potato, tomato is the second most important vegetable fruit crop that belongs to the Solanaceae family and it is the highest processed crop in the world. Tomato is a rich source of proteins, minerals, carbohydrates, and vitamins, especially vitamin C. Tomato also contains many phytochemicals like carotenes and lycopenes which have anticancer properties and other health benefits (Krishna et al., 2021a,b; Rai et al., 2021). In tomato far salt stress tolerance, osmoprotectants genes like *BADH-1, ToOsmotin, Ectoine (ectA, ectB, and ectC)*, and *coda* gene have been transformed in tomato, which reduces the impact of salt stress by encoding osmoprotectant solutes (Moghaieb et al., 2000, 2011; Goel et al., 2010; Wei et al., 2017). To maintain the cellular acidity under salt stress many Na^+^/H^+^ antiporter genes also have been transformed like *NHX1, TaNHX2*, and *LeNHX4* which regulate the Na^+^/H^+^ to maintain cellular homeostasis (Zhang and Blumwald, 2001; Yarra et al., 2012; García-Abellan et al., 2014). Transgenes like *cAPX, MdSOS2L1, AnnSp2, LeNHX2 and SlSOS2, At FeSOD*, and *BcZAT12* have been also transformed and works with different modes of action, details of the gene transformed in tomato and their mode of action are summarized in Table 1. Like potato and tomato, other important Solanaceous crops like brinjal and chili face the salt stress; in these crops also transgenics have been developed for salt stress, and details of the transgenic crop in Solanaceae family are given in Table 1. In vine crops group like cucumber, cucurbits, and bottle gourds are also a very popular in vegetable crops and play an important role in food and nutritional security, salt significantly reduced the yield and quality of vine crops also (Park et al., 2014; Kim et al., 2015; Sun et al., 2018; Li et al., 2020). In water melon *HAL1* transferred which encodes for 32 kDa water soluble proteins which protects from salt induced osmotic stress (Bordas et al., 1997). Park et al. (2014), Han et al. (2015), and Kim et al. (2015) transformed bottle gourds with *AVP1* which encodes vacuolar H^+^-pyrophosphatase, which regulate the proton pump and ultimately maintains the cellular acidity to avoid salt stress (Table 2). Cole crops like cabbage, cauliflower, mustard green, rape seed, and Chinese cabbage constitute a major group of leafy vegetable, which are considered as cheapest and richest sources of mineral, vitamins, and oils and they play an important role in nutrition and food security (Wang et al., 2010; Kim et al., 2016; Ahmed et al., 2017; Luo et al., 2017). The cole crops are very sensitive to the salt stress, and different genes like *CodA*, *PgNHX1*, *OsNASI, BnSIP1-1, APX, SOD, and LEA4-1* have been transferred to sustain salt stress (Park et al., 2005; Wang et al., 2010; Kong et al., 2011). Details of the gene transferred and their mechanism of action in cole crops is given in Table 3.

**TABLE 1:** Transgene used for development of salt stress tolerance, their function and mechanism of action.

S. N.	Genes	Function	Mechanism of action	References
**Potato (*Solanum tuberosum*)**				
1	*P_5_CS*	Encodes for pyrroline-5-carboxylate synthetase (P_5_CS)	*P_5_CS* gene expression enhances proline content in the cells, proline is a potential osmolyte and tolerate to salt stress	Karthikeyan et al., 2011
2	*StCYS1*	Encodes for, Cysteine protease inhibitors (CPI)	Cysteine protease inhibitors (CPI) is a cystatin protein superfamily and facilities biological activities by cysteine protease inhibition	Liu et al., 2020
3	*Glycinebetaine*	Glycinebetaine (GB) synthesizing enzymes	Glycinebetaine GB is a osmolytes and potent compatible compound, its accumulation does not hamper plants normal activities and help in salt tolerance	Ahmad et al., 2014
4	*StDREB1*	Transcription factors	Regulate differential gene expressions in the different signaling pathways due to their different DNA-binding specificity	Bouaziz et al., 2013
5	*AcBADH*	Encodes for *betaine aldehyde dehydrogenase*	Betaine aldehyde dehydrogenase converts betaine aldehyde to glycine betaine which predominantly accumulate in the leaves and stems in dicot and monocot and enhance salt tolerance	Ali et al., 2020
6	*AtNHX1*	Na^+^/H^+^ antiporters	*AtNHX1* gene improves the absorption and transportation of the Na^+^ of the host plant species and enhances salt stress tolerance	Wang et al., 2013
7	*mtlD*	Encodes for mannitol 1-phosphate dehydrogenase	Mannitol accumulation increases in plants in response to osmotic stresses like salt	Rahnama et al., 2011
8	*AtHKT1*	Facilitates high-affinity potassium transporter	HKTs actively involve at the plasma membrane level, HKT transporters exclude Na^+^ from the leaves while increasing K^+^ transportation to resist salt stress	Wang et al., 2019
9	*PaSOD* and *RaAPX*	Encodes for superoxide dismutase (SOD) and ascorbate peroxide (APX) enzymes	SOD and APX enzyme system converts superoxide radical to hydrogen peroxide (H_2_O_2_), followed by conversion of H_2_O_2_ to water and oxygen, respectively	Shafi et al., 2017
10	Rat *GLOase*	Over-expressing L-gulono-c-lactone oxidase	Enhanced ascorbic acid accumulation have been reported to have salt/osmotic stress	Upadhyaya et al., 2010
11	*GalUR*	L-gulono-1,4-lactone conversion to AsA	D galacturonic acid reductase (GalUR over-expression enhances AsA production enhances salt tolerance)	Upadhyaya et al., 2009
12	*GalUR*	Encodes for D galacturonic acid reductase	Overexpression of GalUR, an ascorbate pathway enzyme enhances its ascorbic acid content (L-AsA) and enhances salt tolerance	
13	*StNAC2*	Regulates NAC transcription factors	NAC proteins are plant-specific TFs and to play important roles in abiotic biotic stresses	Xu et al., 2014
14	*AtBADH*	Encodes for betaine aldehyde dehydrogenase	Converts betaine aldehyde to glycine betaine, the elevated glycine betaine level enhances cellular buffering capacity and stress tolerance	Zhang et al., 2011
**Tomato (*Solanum lycopersicum*)**				
1	*BADH-1*	Over expression of betaine aldehyde dehydrogenase	Betaine aldehyde dehydrogenase catalyzes conversion of betaine aldehyde into glycine betaine which improves abiotic stresses tolerance	Moghaieb et al., 2000
2	*NHX1*	Over expression the *NHX1* antiporter	Over expressed *NHX1* vacuolar Na^+^/H^+^ antiporter helps in maintaining cellular integrity and improve salt stress tolerance	Zhang and Blumwald, 2001
3	*cAPX*	Over expression of *APX*	Enhanced activity of ascorbate peroxidase activity reduces cellular damage by scavenging the superoxides under salt stress	Wang et al., 2005
4	*CaKR1*	Over expression of *LeSOD2, LeAPX2*, and *LeAPX3*	High transcript level of antioxidative enzyme machinery scavenge the ROS under abiotic stresses	Seong et al., 2007
5	*ToOsmotin*	Osmotic adjustment	Over expression leads accumulation or compartmentalization of solutes and also protect proteins denaturation under salt stress	Goel et al., 2010
6	*Ectoine (ectA, ectB and ectC)*	Compatible solute	Enhance peroxidase activity and decrease MDA contents by ectoine accumulation	Moghaieb et al., 2011
7	*AtSlSOS2 (AtSlSOS2)*	Homeostasis of Na^+^ and K^+^	Upregulation of the plasma membrane Na^+^/H^+^ (*SlSOS1*) and endosomal-vacuolar K^+^, Na^+^/H^+^ (*LeNHX2* and *LeNHX4*) antiporters, responsible for Na^+^ extrusion out of the root, active loading of Na^+^ into the xylem, and Na^+^ and K^+^ compartmentalization	Huertas et al., 2012
8	*TaNHX2*	Na^+^/H^+^ antiporter	Na^+^/H^+^ antiporters are involved in intracellular ion (Na^+^), pH regulation and K^+^ homeostasis in plants under salt stress	Yarra et al., 2012
9	*HAL5*	Maintaining Na^+^/K^+^ homeostasis	Maintenance of Na^+^ and K^+^ transporters like SlHKT1;2 and SlHAK5 improve homeostasis	García-Abellan et al., 2014
10	*MdSOS2L1*	Codes for *MdSOS2L1* protein kinase	MdSOS2L1 protein kinase physically interacts with MdCBL1, MdCBL4, and MdCBL10 proteins to increase tolerance against salt	Hu et al., 2016
11	*coda*	Encode for glycine betaine	Glycine betaine enhanced NaCl-induced expression of genes encoding the K^+^ transporter, Na^+^/H^+^ antiporter, and H^+^-ATPase	Wei et al., 2017
	*AnnSp2*	Encodes annexins proteins	AnnSp2 alleviated ABA sensitivity in tomato in the germination and seedling stages under salt stress	Ijaz et al., 2017
12	*SlCMO*	Choline monooxygenase (CMO)	Is a key enzyme involved in the synthesis of glycine betaine, which is a osmoprotectant that plays an important role in plant salt tolerance	Li Q. L. et al., 2018
13	*LeNHX2 and SlSOS2*	Homeostasis of Na^+^ and K^+^ and Na^+^/H^+^ antiporter	Involves Na^+^ and/or K^+^ intracellular accumulation mediated by NHX transporters	Baghour et al., 2019
14	*SlMYB102*	Decrease the transcripts of ABA-dependent genes	Suppress the expression of PP2Cs or protein phosphatases of PP2Cs to help plants adapt to higher salt concentrations	Zhang et al., 2020
15	*At FeSOD*	Encodes for super oxide dismutase enzyme	The main function of these enzymes is the enzymatic conversion of such a highly toxic molecule for cells as superoxide into hydrogen peroxide (H_2_O_2_)	Bogoutdinova et al., 2020
16	*LeNHX4*	K^+^, Na^+^/H^+^ antiporter	An important mechanism to overcome salt stress is the exclusion of Na^+^ from the cytoplasm, by the operation of Na^+^/H^+^ antiporters at the plasma membrane or tonoplast. Plant NHX antiporters play a key role in NaCl tolerance by the extrusion of Na^+^ out of cytosol	Maach et al., 2020
17	*COMT1*	Promote the synthesis of melatonin	*SlCOMT1* overexpression could maintain the balance of Na^+^/K^+^ and decrease ion damage by activating salt overly sensitive (SOS) pathway under salt treatment	Sun et al., 2020
18	*BcZAT12*	Encodes for C_2_H_2_ type zinc finger protein	The C_2_H_2_ type zinc finger protein is known to confer tolerance to dehydration, heat stress, salt and/or cold stresses	Rai et al., 2021
**Brinjal (*Solanum melongena*)**				
1	*Yeast HAL1*	Encodes a water**-**soluble protein	*HAL1* and *HAL3*, which were involved in the regulation of K^+^ and Na^+^ transport, respectively, and considerably enhanced salt tolerance in egg plants	Kumar et al., 2014
2	*TaNHX2*	Vacuolar Na^+^/H^+^ antiporter	Na^+^/H^+^ antiporters are involved in intracellular ion (Na^+^), pH regulation, and K^+^ homeostasis in plants	Yarra and Kirti, 2019
3	*adc*	Biosynthetic of polyamine by arginine decarboxylase	Accumulation of higher polyamine in cells works as a osmoprotectants	Prabhavathi and Rajam, 2007
4	*mtlD*	Mannitol-1-phosphate dehydrogenase	The accumulation of mannitol in the cytoplasm and increased tolerance to salt stress	Prabhavathi et al., 2002
**Chili pepper (*Capsicum annuum* L.)**				
1	*TaNHX2*	Vacuolar Na^+^/H^+^ antiporter	Na^+^/H^+^ antiporters are involved in intracellular ion (Na^+^), pH regulation, and K^+^ homeostasis in plants	Bulle et al., 2016
2	*PDH45*	Encodes for Pea DNA Helicase 45	DNA and RNA helicases have proved their translational efficacy in multiple crops by improving tolerance to salinity and drought stress. DNA and RNA helicases, also known as molecular motors, are involved in myriad cellular processes of protein turnover and protection	Shivakumara et al., 2017
3	*Osmotin*	Encodes for Osmotin is a stress-responsive protein	Osmotin is a stress-responsive protein adapted to salinity and desiccation and accumulates in saltadapted cells. Osmotin is an abundant cationic 26-kDa protein that belongs to the family of PR-5 type proteins. Osmotin provides osmotolerance to plants probably by facilitating the compartmentation of solutes	Subramanyam et al., 2011

**TABLE 2:** Transgenic crops developed in vine crops for salt stress tolerance.

S. N.	Genes	Function	Mechanism of action	References
**Cucumber (*Cucumis sativus* L.)**				
1	*LOS5*	Encodes a molybdenum cofactor (MoCo) sulfurase	Molybdenum cofactor (MoCo) sulfurase catalyzes the last step of ABA biosynthesis in plants	Liu Z. et al., 2013
2	*HAL1*	*HAL1* encodes a water soluble protein (32 kDa)	Water soluble protein (32 kDa) that may modulate monovalent ion channels, by affecting the set point of intracellular potassium determined by the feedback regulation of the uptake system	Bordas et al., 1997
3	*CsbHLH041*	Encodes Basic helix-loop-helix (bHLH) transcription factors	The bHLH genes are involved in processes such as metabolic regulation, plant growth and development, and response to environmental signals	Li et al., 2020
	*CmHKT1;1*	Encodes a Na^+^ preferential transporter	(HKT1) encodes a Na^+^ preferential transporter that principally controls root-to-shoot Na^+^ delivery via the withdrawal of Na^+^ from the xylem sap	Sun et al., 2018
4	Bottle gourds			
5	*AVP1*	Encodes vacuolar H^+^-pyrophosphatase	A vacuolar H^+^-pyrophosphatase encoded by the *AVP1* gene is one of the proton pumps in *Arabidopsis* and generates an H^+^ electrochemical gradient across the tonoplast	Kim et al., 2015
6	*AVP1*	Encodes vacuolar H^+^-pyrophosphatase	A vacuolar H^+^-pyrophosphatase encoded by the *AVP1* gene is one of the proton pumps in *Arabidopsis* and generates an H^+^ electrochemical gradient across the tonoplast	Park et al., 2014
	*AVP1*	Encodes vacuolar H^+^-pyrophosphatase	A vacuolar H^+^-pyrophosphatase encoded by the *AVP1* gene is one of the proton pumps in *Arabidopsis* and generates an H^+^ electrochemical gradient across the tonoplast	Han et al., 2015
**Watermelon [*Citrullus lanatus (Thunb*.) Matsum. & Nakai]**				
	*HAL1*	A vacuolar Na^+^/H^+^ antiport	Water soluble protein (32 kDa) that may modulate monovalent ion channels, by affecting the set point of intracellular potassium determined by the feedback regulation of the uptake system	Ellul et al., 2003

**TABLE 3:** Transgenic crops developed in cole crops for salt stress tolerance.

S. N.	Genes	Function	References
**Mustard green (*Brassica juncea*)**			
1	*CodA*	Choline oxidase	Prasad et al., 2000
2	*PgNHX1*	Vacuolar Na^+^/H^+^ antiporter	Rajagopal et al., 2007
3	*AtLEA4-1*	LEA4 protein	Saha et al., 2016
4	*Lectin*	Induced fungal resistance	Kumar et al., 2015
5	*Gly I*	Detoxification of methylglyoxal	Rajwanshi et al., 2016
6	*Gly II*	Detoxification of methylglyoxal	Saxena et al., 2011
	*AnnBj2*	Upregulated expression of ABA-dependent (RAB18) and ABA independent (DREB2B) genes	Ahmed et al., 2017
**Rape seed (*Brassica napus*)**			
	*CodA*	Choline oxidase	Huang et al., 2000
	*AtNHX1*	Vacuolar Na^+^/H^+^ antiporter	Zhang et al., 2001
	*YHem1*	Accelerate endogenous 5-ALA metabolism	Sun et al., 2015
	*LEA4-1*	LEA4 protein	Dalal et al., 2009
	*OsNASI*	11 proteins upregulated including dehydrogenase, GST, POD and Rubisco	Kong et al., 2011
	*DREB*	Expression of many stress-inducible genes	Qamarunnisa et al., 2015
	*BnSIP1-1*	Regulates BnABI5, BnNAC485 or other stress-related genes	Luo et al., 2017
	*PR10*	Pathogenesis related	Srivastava et al., 2004
**Chinese cabbage (*Brassica campestris* L. spp. Chinensis)**			
	*CodA*	Choline oxidase	Wang et al., 2010
**Cabbage (*Brassica campestris*)**			
	*LEA4-1*	LEA protein	Park et al., 2005
**Cauliflower (*B. oleracea* var. botrytis)**			
	*APX, SOD*	Antioxidants	
**Napa cabbage (*Brassica rapa* ssp. Pekinensis)**			
	*BrGI*	Reduced expression of GI, enhanced salt tolerance	Kim et al., 2016

## Transcriptional Regulation of Salinity Stress Signaling in Vegetables

**FIGURE 2:**
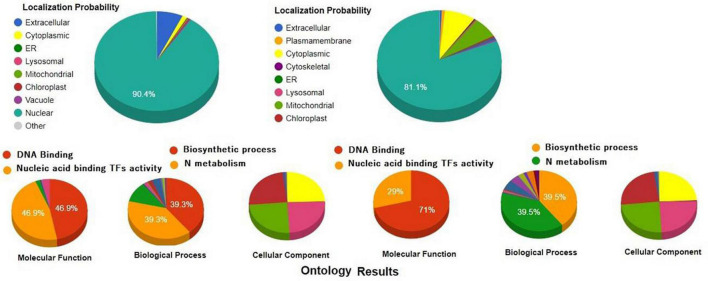
Functional annotation of *WRKY* and NAC proteins. These functional annotation were retrieved from Cello2GO server and were structured around gene ontologies like biological process, molecular function, and cellular component along with their localization probability. It is to be noted that both WRKY and NAC proteins have diversified function with more specifically targeted to DNA binding or Nucleic acid binding transcriptional factor activity.

Recently, it was demonstrated that during salt stress, the expression level of multiple TFs increases much more compared with their basal trends, which reflects their crucial role in regulating the function mechanism and stress-dynamics of stress-tolerance (Franzoni et al., 2019, 2020). TFs are key regulators that play important roles in various stress responses (Debbarma et al., 2019). In fact, TFs are the key players that actually bind with the *cis* acting elements to regulate the spatial and temporal expression of specific genes, or genes regulating the functional activities of signal transduction and/or other genes regulating the transcriptional efficiency of stress-responsive genes under environmental stresses (Liu et al., 2014). Therefore, transcriptomic characterization for identification of stress-responsive TFs in plants or vegetable crops could be useful as a prominent tool or may provide a genetic resource for transgenic technology to improve the stress-responsive traits in different crops (Hong et al., 2021). The TFs belonging to WRKY, NAC, bZIP, MYB, and AP2/ERF play a crucial role in modifying and fine-tuning of the different stress-responsive genes involved in stress avoidance (Golldack et al., 2014). We have provided a comparative pie chart showing the functional annotation of two different transcriptional factors WRKY and NAC having WRKY and NAC domain. Based on functional annotation and gene ontology structured around three ontological terms, biological processes, molecular function, and cellular component, we reported and confirmed the function of WRKY and NAC TFs as to bind with DNA and also playing an important role in metabolism, stress response, and nucleic acid binding transcriptional factor activity (2) (Figure 2). During the last few years, many TFs have been deployed for transgenic overexpression of different TFs to mitigate various abiotic stresses (Tran et al., 2010). For example, the overexpression of moso bamboo *WRKY* (*Phyllostachys edulis) in Arabidopsis* uncovered the importance of *PeWRKY83* in imparting salinity tolerance in transgenic *Arabidopsis* (Wu et al., 2017). SlAREB1, a bZIP transcriptional activator that belongs to ABA-responsive element binding protein (AREB)/ABA-responsive element binding factor (ABF) subfamily overexpression in tomato lines, reported enhanced salt and drought tolerance (Orellana et al., 2010). Furthermore, with the help of CRISPR/Cas9 genome editing technology it has now become possible to edit specific transcriptional factors that could be directly or indirectly fine-tune the expression and regulation of stress-responsive genes against salinity tolerance in plants (Debbarma et al., 2019). For example, CRISPR/Cas9 mediated genome editing of *SlMAPK3* gene in tomato affected the expression level of other drought stress-responsive genes, particularly, *SlDREB, SlLOX*, and *SlGST* in tomato. The downregulation of these genes indirectly affected the salinity response and provided tolerance to salinity. Moreover, genetic engineering, gene silencing, CRISPR/Cas9 mediated-genome editing, transgenic overexpression, gene complementation and genetic transformation, mutant analysis studies done so far for engineering better salt-tolerance strategies in various vegetable and horticultural crops have of course identified novel signaling pathways, interconnected networks, transcriptional activators in mitigating salinity as well as other environmental stresses. Recently, CRISPR/Cas9 technology has provided a novel platform for precise editing of alleles that could assist in providing stress tolerance in plants. Further, the latest advancement in CRISPR-Cas system has sparked the genome editing revolution in plant genetics and breeding. We have discussed the role of some of the important TFs that regulate the stress-tolerance mechanism in plants.

### WRKY Gene Family in Salt Tolerance in Vegetables

*WRKY* gene family is one of the most important transcriptional regulators that regulates stress tolerance mechanism in plants. Many studies done till so far have highlighted the functional role of WRKY gene signaling against various abiotic and biotic stress response in plants (Aamir et al., 2017, 2018, 2019; Hichri et al., 2017; Bai et al., 2018; Li et al., 2020). It has been well documented that *WRKYs* gene-mediated plant defense is controlled by both crossregulation and autoregulation, and extensive signaling involving multiple protein partners like histone acetylases, MAP kinases, MAP kinases kinases (MAPKK), calmodulin, 14-3-3 proteins, and other associated WRKYs partners in a complex network and dynamic web with built in redundancy to fine tune the transcriptional reprogramming, genetic-expression, and stress-tolerance (Rushton et al., 2010). WRKYs prominent role in both abiotic as well as biotic stress tolerance is well-documented (Rushton et al., 2010; Phukan et al., 2016; Bai et al., 2018). WRKYs role in salinity tolerance against various horticultural crops and other plants is well reported (Table 4). For example, Kashyap et al. (2020) reported the relevance of tomato *WRKY1*, *WRKY3*, and *WRKY72* in mitigating salt stress in wild tomato *Solanum chilense* as the expression of these *WRKYs* was more prominent and increased the expression in wild genotype compared with domestic and cultivated genotype DVRT1. Villano et al. (2020) characterized the list of putative WRKYs involved in various abiotic and biotic stresses in two wild relatives of potato *Solanum commersonii* and *Solanum chacoense*, and revealed the ScWRKY23 as multiple stress regulator WRKY in wild potato (Villano et al., 2020). Likewise, Hichri et al. (2016) also reported the expression of tomato *WRKY3* in alleviating salt stress (Hichri et al., 2016). Transcriptomic characterization and function validation through qRT-PCR analysis unraveled the expression profiling and importance of sweet potato WRKYs after treatment with 150 mM salt stress (Qin et al., 2020). In one work, Yue et al. (2019) provided the genome-wide identification and characterization of 92 *WRKY* genes in *Chenopodium quinoa*, and reported the importance of 25 WRKYs in both development and stress tolerance. Similarly, genome-wide identification and characterization of WRKYs in *Cucumis sativus* (cucumber) unraveled the importance of *CsWRKY9*, *CsWRKY18*, *CsWRKY48*, and *CsWRKY57*, in both heat and salt stress tolerance (Chen et al., 2020). In another study, based on Illumina RNA-seq transcriptomic studies, Tang et al. (2014) reported the tissue-specific and differential expression profiles of the *Brassica rapa ssp. pekinensis* (Chinese cabbage) and further validated their role in different abiotic and biotic stresses (Tang et al., 2014). Karanja et al. (2017a) reported 126 WRKYs in *Raphanus sativus* out of which 35 WRKYs had differential expression in various abiotic stresses. Further, the relevance of *WRKY3* in salt tolerance could be better understood as CRISPR/Cas9-mediated *WRKY3* and *WRKY4* mutagenesis in *Arabidopsis*, decreasing both MeJA stress as well as decreased salt tolerance in *Arabidopsis* (Li et al., 2020).

**TABLE 4:** Transcriptional regulation and their mode of action in vegetable crops for salt stress tolerance.

S. No.	Transcription Factor/Gene/Protein	Vegetable	Functional aspects	References
1.	*JUNGBRUNNEN1* (*JUB1*), a NAC transcription factor	*Solanum lycopersicum* (tomato)	Overexpression of *JUNGBRUNNEN1* (*JUB1*) increases salinity tolerance in tomato	Alshareef et al., 2019
2.	BZR/BES transcription factor	*S. lycopersicum* (tomato)	BRASSINAZOLE RESISTANT 1 (BZR1) and BRI1-EMS-SUPPRESSOR 1 (BES1) homologs in have potential role in salt tolerance. SlBZR1D played positive role in salt stress tolerance	Jia et al., 2021
3.	Wild tomato WRKY1, WRKY3, and WRKY72	*S. chilense* (wild tomato)	Salinity stress tolerance	Kashyap et al., 2020
4.	Transgenic overexpression of tomato ERF84 in Arabidopsis	*Arabidopsis thaliana*	Salt and Drought	Li Q. L. et al., 2018
5.	Transgenic overexpression of tomato ERF1	*S. lycopersicum*	Salt Stress	Lu et al., 2011
6.	Microarray analysis for salt-tolerant genes in wild tomato uncovered putative 5ERFs in alleviating salt stress	*S. pimpinellifolium*	Salt stress tolerance	Yang et al., 2018
7.	*ScbZIP* and *SlbZIP*	*S. chilense* (wild tomato) and *S. lycopersicum*	Salinity stress tolerance	Zhu et al., 2018; Kashyap et al., 2020
8.	Tomato SRN1 (*Solanum lycopersicum* stress-related NAC1) plasma membrane-localized protein with transactivation activity in yeast	*S. lycopersicum*	Positively regulates defense response against biotic stress but negatively regulates abiotic stress response	Liu et al., 2014
9.	Tomato NAC35	*S. lycopersicum*	Induced by drought stress, salt stress, bacterial pathogen, and signaling molecules	Wang et al., 2016
10.	Tomato SlAREB1, a bZIP transcription factor, member of the ABA-responsive element binding protein (AREB)/ABA-responsive element binding factor (ABF) subfamily	*S. lycopersicum*	Salt stress and Drought stress tolerance	Orellana et al., 2010
11.	Tomato NAC4 and NAC35	*S. lycopersicum*	Salt, Drought tolerance Biotic stress	Zhu et al., 2014; Wang et al., 2016
12.	ZFP179, a salt responsive gene encoding a Cys2/His2 zinc finger protein	*Oryza sativa*	Overexpression of ZFP179 provided salt tolerance	Sun et al., 2010
13.	BnaABF2, a bZIP transcription factor	*Brassica napus*	Salt tolerance in Transgenic Arabidopsis	Zhao et al., 2016
14.	Chili N*AC46*	*Capsicum annum*	Salt tolerance in transgenic Arabidopsis	Ma et al., 2021
15.	Tomato *DREB2*	*S. lycopersicum* and *A. thaliana*	Salt tolerance	Hichri et al., 2016
16.	Tomato *ERF84, ERF5*	*S. lycopersicum*	Positive regulation for Salt and drought tolerance; negative regulation for biotic stress	Pan et al., 2012; Li Q. L. et al., 2018
17.	Chenopodium *WRKY*	*Chenopodium quinoa*	Stress tolerance and development	Yue et al., 2019
18.	*CsWRKY9, CsWRKY18, CsWRKY48 and CsWRKY57*	*Cucumis sativus*	Heat and salt stress tolerance	Ling et al., 2011; Chen et al., 2020
19.	Radish WRKY	*Raphanus sativus*	Abiotic Stress tolerance	Karanja et al., 2017a
20.	Carrot WRKY20	*Daucus carota*	*DcWRKY20* made interaction with DcMAPK1 and *DcMAPK4* Abiotic and biotic stress tolerance	Li et al., 2016
21.	Tomato NAC1; NAC3	*S. lycopersicum*	Salt stress tolerance; NAC3 suppressed by salt stress	Yang et al., 2011; Han et al., 2012, 2014
22.	Illumina RNA-seq transcriptomic studies of root, stem and leaves in Chinese cabbage	*Brassica rapa ssp. pekinensis*	Abiotic and biotic stress tolerance	Tang et al., 2014
23.	Carrot WRKYs in hormonal regulation and mechanical injuries	*Daucus carota*	Hormone and mechanical injuries	Nan and Gao, 2019
24.	Transcriptomic studies of sweet potato under salt stress	*Ipomoea batatas*	Salt stress tolerance	Qin et al., 2020
25.	Genome-wide identification and characterization of tomato WRKYs under drought, salt and biotic stress	*S. lycopersicum*	Drought, Salt, and Biotic stress	Huang et al., 2012
26.	Genome-wide identification and characterization of WRKYs in wild potato	*S. commersonii* and *S. chacoense*	ScWRKY045 as multiple stress-responsive regulator	Villano et al., 2020
27.	Identification of biotic-stress responsive WRKY from *Brassica oleracea* var. *italica*	*B. oleracea* var. *italica*	Increased expression of BoWRKy6 against biotic stress	Jiang et al., 2016
28.	Genome-wide characterization of potato WRKYs and expression analysis of potato 22 WRKYs under different stresses	*S. tuberosum*	Increased upregulation of *StWRKY01* and *StWRKY39* under different abiotic stresses. *StWRKY58* had highest expression profile under drought and salt stress	Zhang et al., 2017
29.	Genome-wide identification and characterization of WRKYs in brinjal and Turkey berry	*S. melongena L. S. torvum Sw.*)	Biotic stress response	Yang et al., 2015
30	Tomato SR/CAMTA transcription factors SlSR1 and SlSR3L	*S. lycopersicum*	Negatively regulate disease resistance response and SlSR1L positively modulates drought stress tolerance	Li et al., 2014
31	Radish NAC145	*Raphanus sativus*	Salt, heat and drought stresses	Karanja et al., 2017b
32	Melon NAC14	*Cucumis melo*	Overexpression of *CmNAC14* increased the sensitivity of transgenic *Arabidopsis* lines to salt stress	Wei et al., 2016
33.	Potato NAC proteins StNAC072 and StNAC101; StNAC2	*S. tuberosum*	StNAC072 and StNAC101 are orthologs of known stress-responsive *Arabidopsis* RESPONSIVE TO DEHYDRATION 26 (RD26) involved in abiotic stress tolerance; Overexpression of *StNAC2* in transgenic potato increased salt tolerance	Singh et al., 2013; Xu et al., 2014
34.	Watermelon *WRKY ClWRKYs*	*Citrullus lanatus*	Growth, Development, Biotic and Abiotic stress response	Yang et al., 2018
35.	Wild turnip WRKY (BsWRKYs)	*Brassica rapa*	Biotic and abiotic stress response	Kayum et al., 2015
36.	*BjABR1*, an AP2/ERF superfamily gene, from tuber mustard	*Brassica juncea* v*ar. tumida Tsen et Lee*	Abscisic acid and abiotic stress responses	Xiang et al., 2018
37.	*Arabidopsis* NAC2	*A. thaliana*	Stress response and lateral root development	He et al., 2005
38.	Comparative transcriptome and proteome analysis of salt-tolerant and salt-sensitive genotypes of sweet potato and expression profiling of IbNAC07	*Ipomoea batatas*	Salinity stress tolerance	Meng et al., 2020
39.	Genome wide characterization of WRKY genes in summer squash	*Cucurbita pepo*	Water and salt stress tolerance	Bankaji et al., 2019
40.	Genome-Wide Identification of AP2/ERF transcription Factors in cauliflower	*Brassica oleracea L. var. botrytis*	Salt and drought stress tolerance	Li et al., 2017
41	Genome wide characterization of NAC family in celery and further transcriptomic characterization under salt stress AgNAC47 and AgNAC63 were key player	*Apium graveolens*	Heat, salinity, cold stress	Duan et al., 2020
42	Genome-wide characterization of homeobox-leucine zipper gene family in tomato (*Solanum lycopersicum*)	*S. lycopersicum*	Functional analysis of *SlHDZ34* (III sub-family member)under salinity stress revealed salt stress tolerance	Hong et al., 2021
43	SlMYB02, a R2R3-type MYB transcription factor	*S. lycopersicum*	Salt tolerance	Zhang et al., 2020
44.	*CabZIP25*	*Capsicum annum*	Salt tolerance	Gai et al., 2021
45.	Sweet potato *bZIP IbbZIP1; IbABF4*	*A. thaliana*	Transgenic overexpression of *IbbZIP1* in *Arabidopsis* provided salt tolerance; *IbABF4 imparted* multiple stress tolerance	Wang et al., 2019
46.	Tomato bZIP transcription factor SlAREB	*S. lycopersicum*	Salt tolerance	Hsieh et al., 2010
47.	*SlbZIP38* tomato bZIP transcription factor	*S. lycopersicum*	Negative regulator of drought and Salt Stress Tolerance	Pan et al., 2017
48.	*SlbHLH22* a Basic Helix-Loop-Helix (bHLH) transcription factor in tomato	*S. lycopersicum*	Transgenic over expression imparted high tolerance to both salinity and drought	Waseem et al., 2019
49.	*AtMYB20 Arabidopsis* R2R3-MYB transcription factor	*A. thaliana*	Negatively regulated type 2C serine/threonine protein phosphatases to positively regulate salt tolerance	Xu et al., 2014
50.	*SlMYB102*, R2R3-type MYB gene	*S. lycopersicum*	Transgenic overexpression provided salt tolerance	Zhang et al., 2020

### Ethylene Response Factors

The APETALA2/ethylene responsive factor (AP2/ERF) family of transcription factor is one of the prominent groups of transcriptional activator/regulator during various abiotic and biotic stress responses in plants. ERF group has been further classified or subdivided into the dehydration-responsive element-binding proteins (DREBs). The AP2/ERF families in different plants have been further subdivided based on the presence of double AP2 domain, single AP2 domain, and/or single AP2 domain along with presence of a B3-DNA binding domain (Nakano et al., 2006). The interaction of ERF proteins with DRE/CRT motif and *cis*-acting elements is generally associated with stress-responsive genes and plays a crucial role in mitigation of various environmental stresses. For example, transgenic overexpression of *ERF1-V* (*Haynaldia villosa*) in wheat provided salt tolerance (Xing et al., 2017). Similarly, a ERF gene from wheat (*TaERF3*) overexpression had significant results against salt stress compared with control counterparts in wheat (Rong et al., 2014). In one work, Yang et al. (2018) performed the microarray analysis on salt-tolerant genes in wild tomato lines *Solanum pimpinellifolium* PI365967’ under the effect of salt treatment and reported the increased expression of five ERF genes (*SpERF*). Sequence analysis and transcriptomic characterization of these five *SpERFs* uncovered the crucial seven amino acid residues that were involved in binding with GCC box in the promoter of ethylene responsive genes and were shown to be conserved in all the reported ERFs (Yang et al., 2018). In recent years, ERF TFs have been investigated in depth in various horticultural as well as vegetable crops to enhance the breeding program as well as crop improvement, with respect to various environmental stresses in plants (Table 4). For example, transgenic overexpression of tomato ERF84 (*SlERF84*) in *Arabidopsis* provided resistance against salt and drought. Further, tomato ERFTF (*Sl-ERF.B.3*) expression was found to be decreased/downregulated under salinity stress and drought conditions, whereas it has been found to be upregulated/increased following the exposure to cold, flood, and heat response.

### NAC Transcription Family

**FIGURE 3:**
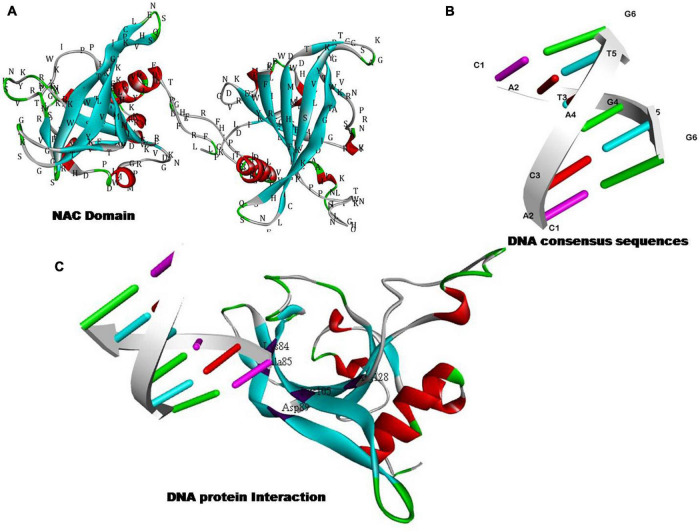
Hypothetical models showing the DNA binding and/or nucleic acid binding transcription factor activity of NAC proteins. **(A)** The structure of NAC domain. **(B)** Structure of NAC binding DNA consensus sequences. **(C)** DNA–protein interaction of NAC proteins with relevant DNA sequences to fine tune the gene regulatory aspects.

NAC (NAM, ATAF, and CUC) TFs have been considered an important group of transcriptional activators that play an important role in developmental programming as well as to encounter challenges against various environmental constraints (Tran et al., 2010; Puranik et al., 2012). The DNA binding property of NAC TFs lie at their N-terminal end (Figure 3). The expression of NAC proteins is highly dependent on the promoter region as each and every NAC gene is characterized by the presence of at least one unique *cis*-element type in their promoter (Li et al., 2016). NAC TFs role in various abiotic and biotic stress responses is well-documented (Table 4). For example, salt-tolerance in tomato is well regulated by NAC1 transcription factor as the enhanced expression of SlNAC1 in root, flower, seeds, and green fruits following the salt stress are well known (Yang et al., 2011). Transcriptomic characterization unraveled the importance of 10 *NAC* genes in tomato against abiotic stresses (Song et al., 2015). In this context, Liu et al. (2014) reported the relevance of tomato NAC transcription factor *SlSRN1* in mediating the positive defense response against biotic stresses while regulating negatively to abiotic stress signaling (Liu et al., 2014). Yang et al. (2011) reported the expression profiling of tomato *NAC1* (*SlNAC*1), an ATAF subfamily transcription factor in different tissues (root, leaves, seeds, and fruit) under salt stress (Yang et al., 2011). Likewise, potato NAC genes StNAC072 and StNAC101 that have been reported as orthologs of known stress-responsive *Arabidopsis* responsive to dehydration 26 (RD26) were found to play a crucial role in mitigating abiotic stress response (Singh et al., 2013). Wei et al. (2016) performed the genome-wide characterization of NAC transcription factor family in melon (*Cucumis melo L*.) and evaluated their expression profile during salt stress. Further, transgenic overexpression of *CmNAC14* in *Arabidopsis* resulted in increased salt-tolerance (Wei et al., 2016). Karanja et al. (2017b) reported the tissue-specific expression profiling of radish NAC TFs and reported the positive regulation of RsNAC023 and RsNAC080 toward all types to abiotic stresses. Further, RsNAC145 had much more active expression profile under salt, heat, and drought stresses when compared with other genes that were expressed under different abiotic stresses (Karanja et al., 2017b). Li et al. (2016) investigated and characterized the list of putative NAC TFs in water melon (*Citruluslanatus*) across the genome and also checked the expression profile and potential function of several NAC TFs in different stresses. Overall, transgene overexpression of *IbNAC7* in *Arabidopsis* provided salt tolerance. Recently, Duan et al. (2020) provided the genome-wide characterization of NAC gene family in leafy vegetable *Apium graveolens* and studied the characterized WRKYs for their stress-tolerance attribute. It was found that a total 111 NAC member were present based on genomic studies. Further, transcriptomic characterization under various abiotic stresses uncovered the *AgNAC63* (ortholog of *ANAC072*/*RD26* role in mitigating salt, cold, and heat stresses). However, the study reported tissue-specific higher expression profiles *AgNAC63* and *AgNAC47* in leaves under the different treatments (Duan et al., 2020).

### Basic Leucine Zipper

The basic leucine (Leu) zipper (bZIP) family also includes one of the most important group of transcriptional activator against abiotic and biotic stress response (Pan et al., 2017). bZIP family also plays an essential role in growth and development of plants (Sornaraj et al., 2016). The bZIP name was based on the presence of the bZIP domain. The bZIP domain is characterized by some specific structural features that is located on an alpha-helix. It has been reported that the first 18 amino acid residues constitute the basic group followed by an invariant N-x7-R/K-x9 motifs for nuclear localization and sequence-specific DNA binding. However, for the second part, the Leu zipper region composed of several heptad repeats of Leu amino acid or other bulky amino acids, such as isoleucine, valine, phenyl nine, tryptophan, or methionine, positioned exactly nine amino acids toward the C-terminus, creating an amphipathic helix (Jakoby et al., 2002; Nijhawan et al., 2008). It has been reported that in bZIP transcriptional proteins, apart from the bZIP domain, some other transcriptional active domains are found and play an essential role in bZIP functioning. The most common site that function as transcriptional activator include phosphorylation site [R/KxxS/T (Furihata et al., 2006; Liao et al., 2008)] and a string of glutamine rich motif. The basic part of the Leu zipper interact with ACGT core region of the B-DNA sequences, particularly, at A-box (TACGTA), G-box (CACGTG), and C-box (GACGTC) (Izawa et al., 1993; Foster et al., 1994). In fact, during the DNA–protein interaction of bZIP proteins with DNA motifs, the half N-terminal of the bZIP domain interacts with DNA major grove region, whereas the other C-terminal end of the Leu zipper constitutes dimer formation for a coiled superimposed structure, defined as zipper superimposed coiled structure, the so-called zipper (Landschulz et al., 1988; Ellenberger et al., 1992). bZIP proteins also play an essential role in imparting tolerance against several abiotic as well as biotic stresses (Table 4). For example, Gai et al. (2021) characterize the list of different bZIP TFs in pepper and reported the relevance of *CabZIP25* in imparting salt tolerance as overexpression done in transgenic *Arabidopsis*. Wang et al. (2018) identified the list of 54 and 50 *bZIP* proteins from whole-genome sequences of *Vigna radiata* and *Vigna angularis*, respectively.

## Grafting Strategies in Vegetable Crops for Salt Stress Tolerance

Improving the productivity of vegetable crops is a challenge under salt-affected soil or water. Hence, increasing salt tolerance in vegetable crops will have a greater impact in nutritional and economic security, particularly of (semi) arid regions, where salinity in soil and water are widespread (Singh et al., 2020). Traditional breeding programs have been attempted to improve salt tolerance in crop plants (Borsani et al., 2003), but the commercial success is limited due to the trait’s complexity. Currently, major efforts are being directed toward genetic transformation in plants to increase their tolerance, and despite the trait’s complexity, the transfer of a single gene or a few genes has resulted in claims of improved salt tolerance, such as the expression of genes involved in the control of Na^+^ transport (Gaxiola et al., 2001). But, the genetically complicated mechanisms of abiotic stress tolerance, as well as the possibility for adverse side effects make this a challenging task (Flowers, 2004). However, unless a full proof practical and faster breeding tool comes in vogue, a well-proven fast and eco-friendly technique “vegetable grafting” can be deployed to increase tolerance to stresses in vegetables. Vegetable grafting, in fact, has emerged as an efficient tool to sustainably increase vigor and yield of commercial cultivars under challenged growth environment by mechanically attaching with resistant root genotypes.

### Grafting Alleviates Salt Stress

Salinity disturbs dry mass partitioning between vegetative and reproductive organs, whereas grafted plants exhibited less alteration (Parthasarathi et al., 2021). In grafting, some rootstocks may have better performance than the others, though their response may change depending on level of salt concentration in the growth medium (Singh et al., 2020; Bayoumi et al., 2021). Numerous reports have demonstrated the ameliorative response of grafting to salinity stress in cucurbitaceous crops (e.g., melon, watermelon, and cucumber) involving the *Cucurbita* interspecific hybrid rootstocks (Goreta et al., 2008; Rouphael et al., 2012). The agronomic performance of pepper cv. “Adige” under natural salinity condition was clearly evident with 75% higher yield and with 31% lesser fruit damage (blossom end rot) when it was grafted onto a salt-tolerant accession “A 25” as rootstock in comparison with non-grafted control plants (Penella et al., 2016). Eggplant (“SuqiQie”) grafting onto the rootstock of wild eggplant (*Solanum torvum* cv. “Torvum vigor”) provided salinity tolerance by minimizing the yield reduction under saline stress (Wei et al., 2009). In contrary, Chen et al. (2003) found that scion genotypes had a significant impact on the growth of grafted tomato plants, regardless of the salinity of the growing environment, but rootstock had no impact.

### Mechanism of Salt Tolerance in Grafted Plants

Grafting is a reciprocal integrative process; the salt tolerance of grafted plants is influenced by both scion and rootstock (Etehadnia et al., 2008). The positive response of grafting can be attributed to more vigorous and robust root systems, greater efficiency of roots for water and nutrient uptake with efficiency to exclude salt-ions, higher photosynthesis, and better oxidative defense system, hormonal regulations, and osmotic adjustment of the grafted plants as compared with the non-grafted plants (Amaro et al., 2014; Rouphael et al., 2017; Singh et al., 2020).

### Root Characteristics

Root, besides providing physical support and anchor to the plants, plays a crucial role in water and ion uptake and their supply to aerial part that help regulate various plant processes (Kumar et al., 2017). However, the alteration in root characteristics is expected to occur since roots being the foremost plant organ exposed to saline growth medium (Singh et al., 2020), consequently the subsequent effects on water and mineral uptake (Rouphael et al., 2017). The numerous reports claim that grafting onto genetically strong root system can effectively mitigate the effect of salinity on the performance of salt-sensitive scion cultivars (Colla et al., 2013); hence it prompts the emphasis of selecting the vigorous root stock for increasing salt tolerance (Colla et al., 2013; Singh et al., 2020). Salinity depressed shoot and root parameters, but grafted plants of tomato onto potato rootstocks were able to avoid the changes in their growths with balanced partitioning between vegetative and reproductive dry masses (Parthasarathi et al., 2021). Furthermore, the tolerance ability of grafting provided by the rootstocks is often associated with the root morphological characteristics to exclude Na^+^ and/or Cl^–^ under saline medium.

### Regulation of Salt and Mineral Ions

Grafted plants tend to restrain Na^+^ and Cl^–^ ions in their root tissues, preventing them from being translocated to the shoots and leaves in high concentrations. The diverse agronomic responses of grafted plants to salinity in numerous studies were resulted by the differential abilities of root genotypes to regulate the uptake and/or translocation of ions of the salts, and of nutrients, due to their competitive interactions (Rouphael et al., 2017). The ability of rootstocks to minimize toxicity of Na^+^ and/or Cl^–^ by exclusion and/or reduction of Cl^–^ absorption by the roots, as well as the replacement or substitution of K^+^ by total Na^+^ in the foliage has been related to the enhancement of salt tolerance by grafting (Martinez-Rodriguez et al., 2008). In pepper, salt-tolerant wild pepper rootstocks “ECU-973” (*Capsicum chinense*) and “BOL-58” (*Capsicum baccatum* var. *pendulum*) provided salinity tolerance in pepper (“Adige”) plants by controlling Na^+^ and Cl^–^ ions accumulation in shoots (Penella et al., 2015). In spite of maintaining better control over Na^+^ and Cl^–^ accumulation in their shoots, grafted plants were able to maintain higher ratio of K^+^/Na^+^ in grafted cucumber on pumpkin rootstock (Usanmaz and Abak, 2019), and higher K^+^ and Ca^++^ in grafted eggplant (Talhouni et al., 2019). Salinity tolerance in a salt-sensitive cucumber (“Jinchun No. 2”) was enhanced by grafting onto a salt-tolerant pumpkin rootstock (“Chaojiquanwang”); this tolerance mechanism shows the better ability of pumpkin rootstock to exclude Na^+^, and thus lesser amount of Na^+^ ions (−69%) reaches the cucumber shoots (Huang et al., 2013).

### Physio-Biochemical Alterations

The tolerance response of grafting on vigorous rootstock with efficiency to control Na^+^ ion accumulation in shoots has been associated with the efficiency of rootstocks to modulate water uptake by roots and losses by transpiration. Grafting onto some rootstock was useful to maintain better leaf water status than the others. Grafting tomato (“Ikram”) on potato rootstock (“Charlotte”) was found promising to increase salinity tolerance of 5.0 dS/m in grafted tomato with enhanced water productivity (+56.8%) (Parthasarathi et al., 2021). Grafting onto certain rootstocks was able to mitigate salt induced photoinhibition of photosynthesis and consequently growth of grafted plants (Liu Z. et al., 2013). As a coping mechanism of oxidative damage, plants activate enzymatic (i.e., ascorbate peroxidase, catalase, superoxide dismutase, monodehydroascorbate reductase, dehydro ascorbate reductase, and glutathione reductase) as well as non-enzymatic (i.e., reduced glutathione, reduced ascorbate, carotenoids, and tocopherols) antioxidant systems (Rouphael et al., 2017; Singh et al., 2020). Grafting studies demonstrated that some of the graft combinations have a better ability to mitigate salinity stress by regulating antioxidative defense system than the others. Salt-stressed eggplants experienced oxidative stress (higher malondialdehyde, MDA), whereas grafted eggplants were capable of mitigating ROS induced by oxidative stress as a result of increased level of antioxidant enzymes (SOD, CAT, and APX) (Talhouni et al., 2019). Polyamines increase under salinity stress and hence increases plants tolerance; the increased level of polyamines (free, soluble, and conjugated polyamines) provided better tolerance to salinity (i.e., excess calcium nitrate) in grafted seedlings than the non-grafted tomatoes (Wei et al., 2009). Stegemann and Bock (2009) reported that plant grafting can result in the exchange of genetic information *via* either large DNA pieces or entire plastid genomes. However, gene transfer is restricted to the contact zone between scion and rootstock. Thus, the use of rootstock cannot change the sensitivity of scion itself to salt stress. Working with model plant *Arabidopsis*, Shi et al. (2002) reported that SOS1 (salt excessively sensitive) gene is expected to play a role in the loading of Na^+^ into the xylem tracheids from xylem parenchyma cells. Further reports suggest that in *Arabidopsis*, high affinity K^+^ transporters (HKTs) were involved in the removal of Na^+^ from the xylem (Rus et al., 2001, 2006; Sunarpi et al., 2005; Davenport et al., 2007), and hence the leaves were safe from Na^+^ ion toxicity. Furthermore, it was recently discovered that expressing the Na^+^ transporter HKT1;1 in the mature root stele of *Arabidopsis thaliana* utilizing an enhancer trap expression system reduced Na^+^ build up in the shoot by 37–64%, and hence increased salinity tolerance (Møller et al., 2009). Using grafting experiments, it was discovered that HKT1;1 expressed in the root rather than the shoot regulates Na^+^ accumulation in *Arabidopsis* shoots (Rus et al., 2006), implying that the SOS1 analogous gene and HKTs are likely involved in Na^+^ transport in the pumpkin rootstock, allowing it to limit Na^+^ transport from the root to the shoot.

Grafting onto some rootstocks has shown to also increase scion’s tolerance to salinity by modulating the hormonal balance namely of ABA, cytokinins, and polyamines (Rouphael et al., 2017). The reduced transpiration with maintained leaf water relations by elevated level of shoot ABA concentration under salt stress have been reported (Singh et al., 2020). The enhanced salinity tolerance in tomato was related to increased level of ABA content in scion shoots, irrespective of the rootstocks raised under saline condition (Chen et al., 2003). Likewise, increased root-to-shoot cytokine transport by rootstock that overexpressed cytokinin biosynthesis genes (e.g., isopentenyl transferase) was associated with the increased salinity tolerance in tomato presented by maintained stomatal conductance and photosystem II efficiency accompanied with lesser accumulation of toxic ions, consequently producing higher shoot and fruit growths (Ghanem et al., 2011).

## Conclusion

Abiotic stresses like salt stress which persists throughout plant’s whole life cycle negatively affects plant yield and nutritional quality. To ensure vegetable production under salt stress many transgenes have been transferred to the vegetables. Transforming vegetables are the one of the most reliable technique to cope salt stress as most of the vegetable gene pool lack novel gene for salt stress in its gene pool. To manage salt stress at molecular level, the native TFs in the vegetable crops are also being regulated to sustain yield and quality under salt stress. Grafting has shown potential to alleviate salinity stress (water or soil) on the selected vigorous and tolerant rootstocks. Certain wild accessions which possess resistance to salinity but are difficult to introgress these traits into commercial cultivars through traditional breeding tools, can be utilized as rootstock to increase grafted scion’s efficiency or tolerance to salinity. Plant biologists around the world are grappling with the dilemma of exponential population growth and rising food demand. Abiotic stress, such as salt, is a major threat to agricultural productivity and has been linked to worsening food security trends since the beginning. Soil salinity and degradation of soil quality are linked to lower agricultural yields. The production of salinity-tolerant crops is the only way to ensure global food. The actual yield produced by saline soils is more than half of what was originally predicted for normal soils. Organic matter and biodiversity are quite low in these soils.

## Author Contributions

All authors listed have made a substantial, direct, and intellectual contribution to the work, and approved it for publication.

## Conflict of Interest

The authors declare that the research was conducted in the absence of any commercial or financial relationships that could be construed as a potential conflict of interest.

## Publisher’s Note

All claims expressed in this article are solely those of the authors and do not necessarily represent those of their affiliated organizations, or those of the publisher, the editors and the reviewers. Any product that may be evaluated in this article, or claim that may be made by its manufacturer, is not guaranteed or endorsed by the publisher.
